# Hybrid Incompatibilities and Transgressive Gene Expression Between Two Closely Related Subspecies of *Drosophila*

**DOI:** 10.3389/fgene.2020.599292

**Published:** 2020-12-10

**Authors:** Alwyn C. Go, Alberto Civetta

**Affiliations:** Department of Biology, The University of Winnipeg, Winnipeg, MB, Canada

**Keywords:** regulatory co-option, network interactions, speciation, selection, transgressive gene expression, compensatory evolution, gene expression divergence

## Abstract

Genome-wide assays of expression between species and their hybrids have identified genes that become either over- or underexpressed relative to the parental species (i.e., transgressive). Transgressive expression in hybrids is of interest because it highlights possible changes in gene regulation linked to hybrid dysfunction. Previous studies in *Drosophila* that used long-diverged species pairs with complete or nearly complete isolation (i.e., full sterility and partial inviability of hybrids) and high-levels of genome misregulation have found correlations between expression and coding sequence divergence. The work highlighted the possible effects of directional selection driving sequence divergence and transgressive expression. Whether the same is true for taxa at early stages of divergence that have only achieved partial isolation remains untested. Here, we reanalyze previously published genome expression data and available genome sequence reads from a pair of partially isolated subspecies of *Drosophila* to compare expression and sequence divergence. We find a significant correlation in rates of expression and sequence evolution, but no support for directional selection driving transgressive expression in hybrids. We find that most transgressive genes in hybrids show no differential expression between parental subspecies and used SNP data to explore the role of stabilizing selection through compensatory mutations. We also examine possible misregulation through cascade effects that could be driven by interacting gene networks or co-option of off-target *cis*-regulatory elements.

## Introduction

Studies that have addressed the genetic basis of incompatibilities in hybrids between species, or diverging populations, have traditionally resorted to mapping loci and interactions between them (Coyne and Orr, 1989; Masly and Presgraves, 2007; Presgraves, 2008; Victoria Cattani and Presgraves, 2012; Dufresnes et al., 2016). This approach has been fruitful in that ultimately a few major protein-coding genes have been identified (Ting et al., 1998; Masly et al., 2006; Mihola et al., 2009; Phadnis and Allen Orr, 2009), and in some cases, the effect of these major genes require interactions with other genetic factors. Major genes often show patterns of rapid evolution between divergent populations or species (Ting et al., 1998; Presgraves et al., 2003; Maheshwari and Barbash, 2011) suggesting that, at least in part, changes in protein composition might exert effects on phenotype and function through alterations in patterns of expression of genes targeted by such proteins. Moreover, genome-wide surveys have provided evidence to support that many genes and complex systems of epistasis are linked to hybrid incompatibility phenotypes (Morán and Fontdevila, 2014; Turner and Harr, 2014; Turner et al., 2014; Fontdevila, 2016). While co-evolution among interacting genes keeps function within populations and species, hybridization between divergent isolated populations and incipient species brings together incompatible interloci allele interactions resulting in a reduction in hybrid fitness (Dobzhansky, 1937; Muller, 1942; Orr, 1996). The reduced fitness of hybrids serves as a postzygotic barrier among divergent taxa.

The role of divergence in the regulation of gene expression has been long acknowledged (King and Wilson, 1975), but not until recently has genome-wide divergence in gene expression during speciation been addressed. Recent reviews have summarized how changes in gene expression could impact hybrid phenotypes (Civetta, 2016; Mack and Nachman, 2017). Using genome-wide approaches, questions have been addressed as to the proportion of genome-wide misregulation in hybrids, the relative contribution of *cis-* vs. *trans-*regulatory elements in gene misregulation, and the identity of misregulated genes that might contribute to hybrid fitness breakdown (Ranz et al., 2004; Haerty and Singh, 2006; Renaut et al., 2009; Tirosh et al., 2009; McManus et al., 2010; Llopart, 2012; Coolon et al., 2014; Gomes and Civetta, 2015; Brill et al., 2016; Mack et al., 2016). Often, genome-wide assays of expression in hybrids reveal gene regulatory dysfunctions as patterns of transgressive gene expression (i.e., expression beyond levels found in parental species). This can be a consequence of directional selection or drift causing changes at *cis*- and *trans*-regulatory elements that drive divergence in expression between taxa and transgressive expression in hybrids. Previous studies have found positive correlations between protein-coding evolution and gene expression divergence between species of *Drosophila* (Castillo-Davis et al., 2004; Nuzhdin et al., 2004; Lemos et al., 2005; Artieri et al., 2007). Moreover, the finding of a similar significant positive correlation between non-synonymous (d_*N*_) and non-synonymous/synonymous (d_*N*_/d_*S*_) divergence and gene expression differences between hybrids and parental species has been used to suggest sequence divergence driving regulatory incompatibilities and to highlight the potential effects of directional selection in gene expression during speciation (Artieri et al., 2007; Hunt et al., 2013). However, the species pairs used were typically long diverged with hybrids exhibiting complete or nearly complete isolation and high levels of genome misregulation (Ranz et al., 2004; Haerty and Singh, 2006; Artieri et al., 2007; McManus et al., 2010; Coolon et al., 2014). The use of divergent populations within species of copepods have found no significant relationship between hybrid transgressive expression and estimates of sequence divergence, and the authors offered an alternative physiological explanation for the detected pattern (Barreto et al., 2015, 2018).

There are, in fact, alternative explanations that could explain the lack of relationship between sequence and expression divergence. Mutations within taxa can work to compensate the effect of deleterious mutations on expression (i.e., stabilizing selection). The possibility that *cis–trans* mutations may cause compensation within species but lead to transgressive expression in hybrids is supported by studies that report abundant *cis–trans* epistasis (Mackay, 2014; Mackay and Moore, 2014; He et al., 2016; Vonesch et al., 2016). However, the strength of selection for a secondary compensatory mutation might be small (Bourguet, 1999). It is also possible for transgressive expression in hybrids to arise as a response to hybrid dysfunction within gene-interacting networks or metabolic pathways. While this could work to ameliorate fitness problems in hybrids, it could also exacerbate hybrid dysfunction. This might be particularly the case for fitness breakdown between diverging populations (Barreto et al., 2015, 2018). Finally, we speculate that newly arising mutations in *trans* regulatory elements that result from divergence between taxa or compensatory mutations within, could co-opt preexisting *cis*-regulatory elements among multiple genes thereby causing widespread misregulation.

Here, we used a pair of geographically separated subspecies of *D. pseudoobscura*, *D. p. pseudoobscura*, and *D. p. bogotana* that have diverged for at least 0.15 Myr (Schaeffer and Miller, 1991; Wang et al., 1997) and whose hybrids exhibit unidirectional male sterility where only male hybrids produced by *D. p. bogotana* females are sterile. We reanalyze previously published transcriptomics data (Gomes and Civetta, 2015) using a newer *D. p. pseudoobscura* genome release (r3.04) and updated mapping and expression analysis tools to explore relationships between genome expression and gene-coding sequence divergence. Our report identifies no relationship between sequence divergence and transgressive expression in hybrids suggesting a need for broader examinations of transgressive expression between recently diverged populations and species across taxa. We find that most transgressive genes in hybrids are not differentially expressed between subspecies. We explore explanations for transgressive expression other than incompatibilities in regulation arising from rapid divergence between subspecies, such as compensatory mutations, gene-interaction networks, and the co-option of multiple *cis*-regulatory elements by *trans*-regulatory elements. While we find some support for these alternative hypotheses, we acknowledge that they do not fully explain transgressive expression in hybrids. We discuss some caveats and offer other possible explanations in the hope that they will trigger further inquiry. Ultimately, full comprehension of transgressive expression in hybrids will require combining information on genome expression and sequencing with the identification of interactomes and a proper characterization of mechanism of *trans* effects on characterized *cis*-regulatory targets.

## Materials and Methods

### RNA-Sequence Data

Raw RNA sequence data used in this analysis were from a genome-wide transcriptomics study of the *Drosophila pseudoobscura* subspecies pair and their reciprocal hybrids by Gomes and Civetta (2015). Briefly, RNA was extracted from the whole male reproductive tract. Biological replicates were obtained for the parental subspecies and their reciprocal F_1_ hybrids with each replicate containing 30–40 male reproductive tracts. cDNA libraries were prepared using the Illumina TruSeq Stranded mRNA sample preparation kit and multiplexed on a single lane of an Illumina HiSeq2000 platform with 100 bp paired-end sequencing. A quality check was performed on the raw reads using FastQC (Andrews, 2010). Read processing and adapter trimming were performed with Trimmomatic (Bolger et al., 2014), and reads with a Phred score below 30 and a final length of 50 bp were excluded.

### Mapping and Differential Expression Analysis

We mapped processed reads to the latest release (r3.04) of the *D. p. pseudoobscura* reference genome^1^ using STAR, chosen for its reliability (Dobin et al., 2013; Baruzzo et al., 2017) over the previously used TopHat approach (Gomes and Civetta, 2015). Read counting was performed at the gene level using featureCounts (Liao et al., 2014) with the reversely stranded (-s 2) and fragment counting (-p) parameters and the latest version of the *D. p. pseudoobscura* annotation serving as a guide.

Pairwise differential expression across all groups was performed using both DESeq2 (Love et al., 2014) and edgeR (Robinson et al., 2009). In the analysis using edgeR, genes with less than 1 count per million (CPM) in at least one group were excluded from further analysis, and the per gene counts for each sample were normalized using the TMM method (Robinson and Oshlack, 2010). The default settings were used to obtain normalized counts from the DESeq2 analysis. The consensus list of differentially expressed genes from both tools were used for all downstream analyses. Differentially expressed genes among the hybrids were identified as transgressive if their expression were significantly above or below the range found in the parental subspecies. Further, log_2_ fold-changes (lfc) thresholds of 0.5 and 1 were applied to increase our statistical yield of true positives (Schurch et al., 2016). All tools for the analysis were ran on Galaxy^2^.

### Coding Sequence and Expression Divergence

Rates of coding sequence divergence between *D. p. bogotana* and *D. p. pseudoobscura* were estimated for differentially expressed genes between the parental subspecies and for transgressive genes in fertile and sterile F_1_ hybrids. Since the RNA-seq data provided only partial sequences from each gene analyzed, we retrieved raw DNA sequence reads from the sequence read archives (SRA) under the accession number SRX091468 (*D. p. bogotana*). The *D. p. bogotana* raw sequence reads were aligned to all gene regions from the r.3.04 *D. p. pseudoobscura* reference genome (see text footnote 1) using BWA (Li and Durbin, 2010) ran on Galaxy (see text footnote 2) under default settings except for the maximum number of gap extensions, which was set to 4. The “extract consensus from assembly” workflow in UGene (Okonechnikov et al., 2012) was then used to extract the *D. p. bogotana* gene regions, and these were aligned to the longest available transcript for *D. p. pseudoobscura* from FlyBase (see text footnote 1) using MAFFT (Katoh and Standley, 2013). The alignments were modified using Gblocks v.0.91b (Castresana, 2000) with default settings except for the block parameters, which allowed gap positions with half within the final blocks–this removes unaligned introns from the *D. p. bogotana* gene region while preserving possible indels. Alignments from Gblocks were inspected to ensure that the coding sequences were intact open reading frames and were a multiple of three.

Rates of synonymous (d_*S*_) and non-synonymous (d_*N*_) nucleotide substitutions were estimated using the SeqinR package (Charif and Lobry, 2007) loaded on RStudio version 1.1.463. Non-parametric Spearman rank sum correlation coefficients were calculated to test the relationship between coding sequence divergence (d_*N*_, d_*S*_, and d_*N*_/d_*S*_) and expression difference. For the parental subspecies, expression differences were calculated as the absolute difference of [log_2_($\bar{x}$*_*D. p. pseudoobscura*_*) - log_2_($\bar{x}$*_*D. p. bogotana*_*)]. For the transgressive genes, expression differences were calculated for each hybrid relative to each parental subspecies as the absolute difference of [log_2_($\bar{x}$_*Fert or Ster*_) - log_2_($\bar{x}$*_*D. p. pseudoobscura or D. p. bogotana*_*)]. The lower absolute difference value was kept as a measure of minimum transgressive expression (Barreto et al., 2015).

### Allele-Specific Expression

To determine the role of *cis* and/or *trans* changes to transgressive gene expression in the hybrids, we identified fixed species-specific single nucleotide polymorphisms (SNPs) and their relative allele expression in the hybrids. SNPs between the parental subspecies were identified from their mapped reads using Naïve variant caller followed by processing with the Variant annotator (Blankenberg et al., 2014). SNPs were considered fixed in each parental subspecies if each parent had a single different allele and at least three supporting reads. Allele-specific expression (ASE) in the hybrids was measured by first assigning their RNA-seq reads to a parent of origin based on the identity of the allele at fixed SNP positions in each parent. Reads with fixed SNPs mapping to a single gene were summed, and any gene with less than 20 mapped reads from both parental subspecies combined were discarded from further analysis (McManus et al., 2010; Gomes and Civetta, 2015). SNP counts for each gene were then adjusted to account for differences in sequencing depth between samples. Samples with zero SNP counts were given a value of 1 to allow for statistical testing. To detect significant differences between the ratio of parental SNP counts to counts of each parental allele in the sterile and fertile hybrids, respectively, the Fisher’s exact test was used (McManus et al., 2010; Gomes and Civetta, 2015). Transgressive genes that showed differential expression between the parental subspecies were classified as driven by *cis*–*trans* divergence if the Fisher’s exact test was significant and *cis* regulatory divergence when the Fisher’s exact test was not significant (McManus et al., 2010). For transgressive genes that were not differentially expressed between the parental subspecies, a significant result for the Fisher’s exact test indicated evidence for compensatory *cis* and *trans* mutations (McManus et al., 2010), while a non-significant result suggested a conservation in regulatory interactions and classified as non-compensatory.

### Interactions and Sequence Similarity

Interactions among proteins were predicted using STRING (v11.0; Szklarczyk et al., 2019). Gene-Ontology and UniProt keyword enrichments were assessed from outputs using STRING and DAVID (v6.8; Huang et al., 2009a,b). We used the extended gene regions (which includes 2-kb 5′ and 3′) for genes that showed transgressive expression driven by *trans* regulatory elements (i.e., *cis–trans* divergent or compensatory) to perform a BLASTn against a database containing all transgressive genes and against another database with all *D. p. pseudoobscura* extended gene regions within the genome to identify similarities between upstream regions for plus/plus matches or between the upstream and downstream regions for plus/minus matches. We retained only hits that were lower than 1 × 10^–14^ and unique among transgressive sequences and not shared with other genes in the genome. Retained hits had *E*-values lower than 8 × 10^–15^, with nucleotide alignments of at least 173 base pairs and identities higher than 64%.

## Results

The re-analysis of our previously published data (Gomes and Civetta, 2015) by mapping reads onto a newer released genome assembly and using more recently developed analytical pipelines found similar results in terms of lack of bias in mapping, low proportion of differentially expressed genes between subspecies, and significant excess of transgressive expression in sterile relative to fertile hybrids (Supplementary Material).

### Transgressive Gene Expression in Hybrids Does Not Correlate With Accelerated Rates of Evolution as Expected Under a Scenario of Divergent Selection Between Subspecies

**FIGURE 1:**
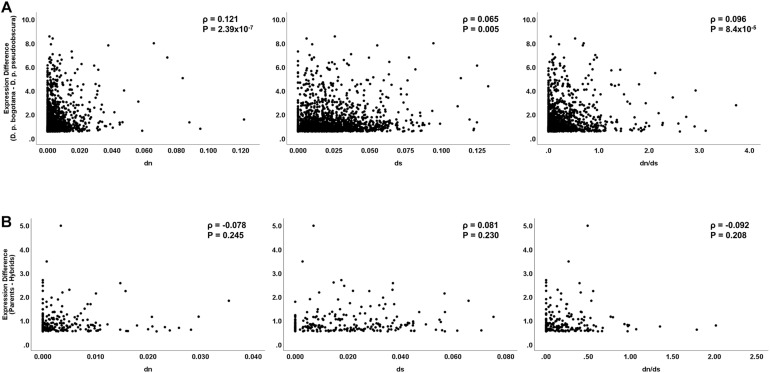
Correlation analysis between expression and coding sequence divergence. Spearman’s rank-sum coefficient and *P*-values are displayed in each frame. **(A)** Analysis on differentially expressed genes between the parental subspecies. **(B)** Analysis on genes showing transgressive expression in hybrids (fertile and sterile).

Under the assumption that regulatory evolution and structural protein evolution are under similar selective pressures, a correlation is expected between expression difference and nucleotide sequence evolution. Of the 819 differentially expressed genes between the parental subspecies, 604 (73.7%) were protein-coding genes with the remaining 215 (26.3%) being non-coding RNAs or coding genes without full coding sequences available for both subspecies. The percentage of differentially expressed protein-coding genes between subspecies increases significantly when a less stringent lfc threshold of 0.5 was applied (82.7%; *Z* = 5.51, *P* < 0.001) (Supplementary Figure 1). We found a significant correlation for expression differences between subspecies and non-synonymous (d_*N*_) sequence divergence (*N* = 604; Spearman’s ρ = 0.091, *P* = 0.026) but not between differences in expression and synonymous substitutions (d_*S*_) (Spearman’s ρ = -0.046, *P* = 0.261). The d_*N*_/d_*S*_ ratio was also positively correlated with expression differences (ρ = 0.108, *P* = 0.011). Using the less stringent lfc threshold of 0.5, d_*N*_, d_*S*_, and d_*N*_/d_*S*_ were all significantly correlated with gene expression divergence between subspecies (*N* = 1,801; ρ = 0.121, *P* = 2.39 × 10^–7^; ρ = 0.065, *P* = 0.005; and ρ = 0.096, *P* = 8.4 × 10^–5^, respectively) (Figure 1A). These results are overall in agreement with previous findings in *Drosophila* and other organisms confirming that protein sequence and expression divergence are influenced by similar selective processes (Castillo-Davis et al., 2004; Nuzhdin et al., 2004; Khaitovich et al., 2005; Artieri et al., 2007; Ortíz-Barrientos et al., 2007).

Given that protein-coding sequence differentiation serves as a good predictor of expression divergence, some studies have explored correlations between rates of protein divergence with expression of misregulated genes in hybrids. Misregulated genes with transgressive expression in hybrids are of interest in speciation as they associate with hybrid disrupted phenotypes (Moehring et al., 2007; Catron and Noor, 2008; Sundararajan and Civetta, 2011; Gomes and Civetta, 2015; Brill et al., 2016; Civetta, 2016). Significant positive correlations are suggestive of either directional selection or relaxation of selective constraints fueling regulatory incompatibilities (Artieri et al., 2007; Hunt et al., 2013; Barreto et al., 2015). Of the 44 transgressive genes in the hybrids, 35 had available sequence data for the estimation of coding sequence divergence. The analysis showed no significant correlations between sequence divergence and expression difference (*N* = 35; d_*N*_, ρ = 0.078, *P* = 0.655; d_*S*_, ρ = 0.242, *P* = 0.161; d_*N*_/d_*S*_, ρ = -0.112, *P* = 0.547). This result holds when a less stringent lfc threshold of 0.5 was used, with 223 of the 262 transgressive genes having sequence data available for analysis (*N* = 223 d_*N*_, ρ = -0.078, *P* = 0.245; d_*S*_, ρ = 0.081, *P* = 0.230; d_*N*_/d_*S*_, ρ = -0.092, *P* = 0.208) (Figure 1B).

### Alternative Explanations for Transgressive Expression in Hybrids: Compensatory Mutations, Interaction Networks, and Transcriptional Drive by Sequence Similarity Among Targets

**FIGURE 2:**
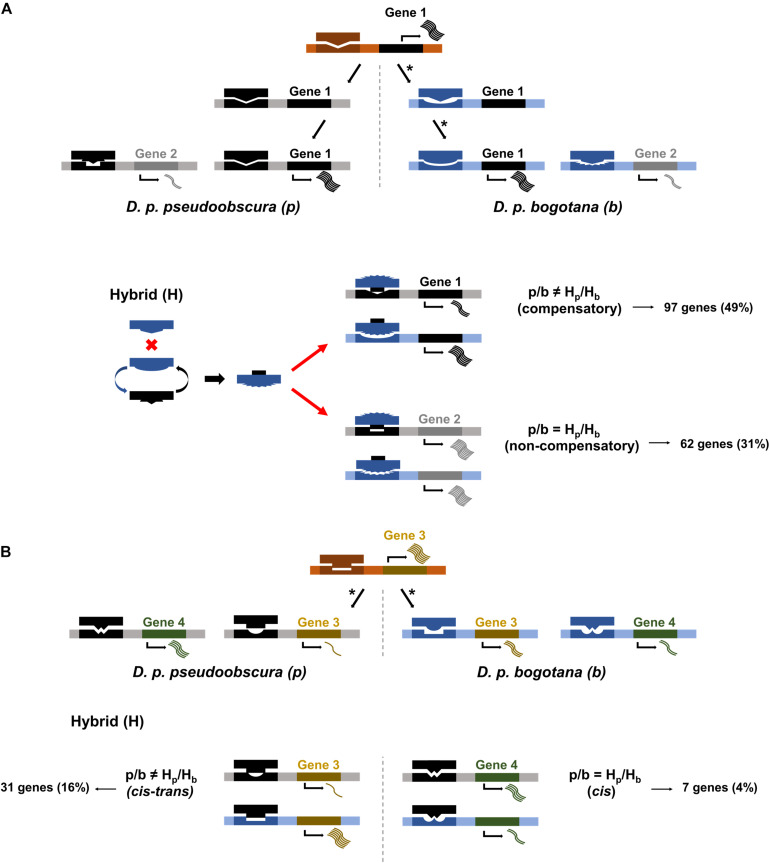
Scenarios of regulatory divergence for *cis* and *trans* regulatory elements. **(A)** Gene 1 shows compensatory *cis* and *trans* mutations, denoted by asterisks, wherein *D. p. bogotana* experiences an initial mutation in *cis* followed by a mutation in *trans* restoring gene expression to similar levels between parental subspecies. Gene 2 shows similar levels of expression in parental subspecies. In the hybrid background, the *D. p. bogotana trans* factor for gene 1 interacts with the *D. p. pseudoobscura trans* factor for gene 2 leading to a conformation change. This new *trans* factor complex can now bind optimally to the *cis* region of genes 1 and 2 (red arrows) resulting in transgressive expression (i.e., expression above parental levels). The allelic ratio of gene 2 in the hybrid is equal to the allelic ratio of the two subspecies, and the gene is classified as non-compensatory through SNP analysis. **(B)** Gene 3 shows divergence in *cis* in one subspecies and *trans* in the other subspecies. This leads to suboptimal binding in both subspecies and differential expression. The regulatory incompatibilities persist within the hybrid background leading to unequal allelic ratios. Gene 3 is classified as *cis–trans* divergent by SNP analysis. Gene 4 shows a situation of *cis*-only divergence between the parental subspecies. Regulatory incompatibilities would occur in *D. p. bogotana* but not *D. p. pseudoobscura* resulting in differential expression between the subspecies. Similar interactions for this gene would occur in the hybrid resulting in equal allelic ratio. Gene 4 is classified as *cis*-only by SNP analysis.

One possibility for a lack of correlation between transgressive expression in hybrids and sequence divergence is that transgressive expression might be a consequence of occasional deleterious mutations that are followed by compensatory DNA changes to overcome detrimental effects on gene expression (i.e., a side effect of stabilizing selection between divergent taxa) (Figure 2A–Gene 1). Our data show that 32 out of 44 (72.72%) transgressive genes in the hybrids were not differentially expressed between parental subspecies. The low number of transgressive genes is likely a consequence of our stringent use of a twofold change (lfc = 1) in expression threshold to maximize our statistical yield of true positives. Given the low sample size, we decided to continue using a less stringent lfc threshold of 0.5 and found, as with the more stringent threshold, a large proportion of transgressive genes without differential expression between parental subspecies (79%, 207/262). If genes without differential expression between subspecies are under stabilizing selection favoring compensatory mutations to buffer deleterious mutations and restore expression to similar levels among parental subspecies, we expect their rate of sequence divergence to be lower than those of genes experiencing divergence in regulation, and thus expression, between subspecies. Our data shows no significantly lower rates of change (d_*N*_ and d_*N*_/d_*S*_) for genes with transgressive expression in hybrids and no differential expression between parentals (Mann–Whitney FDR corrected *P*-values) (Table 1).

**TABLE 1:** Average evolutionary rates (±SD) for differentially expressed genes between parental subspecies that do not show transgressive expression in hybrids [(P_1_ ≠ P_2_)_*NT*_], transgressive genes that show differential expression between subspecies [(P_1_ ≠ P_2_)_*T*_], and transgressive genes that do not show differential expression between subspecies [(P_1_ = P_2_)_*T*_].

	Non-transgressive	Transgressive	
		
	(P_1_ ≠ P_2_)_*NT*_	(P_1_ ≠ P_2_)_*T*_	(P_1_ = P_2_)_*T*_
*N*	1,763	49	174
d_*N*_	5.022 × 10^–3^ (±1.74 × 10^–2^)	4.461 × 10^–3^ (±6.02 × 10^–3^)	4.060 × 10^–3^ (±5.90 × 10^–3^)
d_*S*_	2.290 × 10^–2^ (±2.82 × 10^–2^)	2.086 × 10^–2^ (±1.83 × 10^–2^)	1.890 × 10^–2^ (±1.60 × 10^–2^)
d_*N*_/d_*S*_	2.513 × 10^–1^ (±3.81 × 10^–1^)	2.171 × 10^–1^ (±2.34 × 10^–1^)	2.389 × 10^–1^ (±3.19 × 10^–1^)

*FDR corrected Mann–Whitney tests show no significant differences between rates of non-synonymous substitutions (d_*N*_), synonymous substitutions (d_*S*_), and the d_*N*_/d_*S*_ ratio across all three comparisons.*

We used informative SNPs to identify genes with transgressive expression in hybrids driven by compensatory mutations or *cis*–*trans* divergence (Figures 2A,B–Genes 1 and 3). Twenty-five percent of the transgressive genes (65/262) had non-informative SNPs to allow us to classify parent of origin for the alleles found in the hybrids. Of the remaining 197 transgressive genes, we found that for 65% of them, transgressive expression could be explained by compensatory mutations (97 genes) or *cis–trans* divergence (31 genes) (Figures 2A,B–Genes 1 and 3). The remaining are cases in which the transgressive gene shows similar ratios of subspecies allele expression in parents and hybrids. Of these, 62 were classified as non-compensatory and 7 as having experienced *cis* divergence (Figures 2A,B–Genes 2 and 4).

**FIGURE 3:**
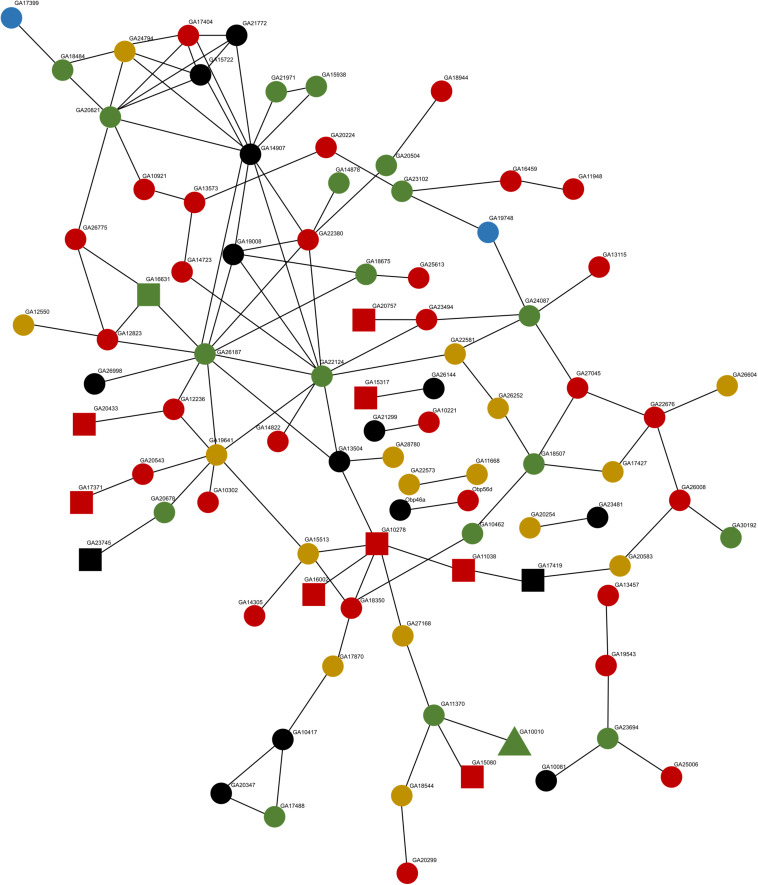
STRING protein–protein interaction network for all transgressive genes in hybrids. Circles represent transgressive genes that are unique to the sterile hybrid (78), squares are genes unique to the fertile hybrid (11), and the triangle represents a gene that shows transgressive expression in both fertile and sterile hybrids. Non-compensatory genes (20) are colored green, red represents compensatory genes (37), yellow for genes with *cis*–*trans* divergence (16), blue for *cis*-only genes (2), and black represents genes with no informative SNPs (15).

We explored whether transgressive expression in hybrids for genes that do not show evidence of compensatory or *cis–trans* mutations could be a cascade triggered by interactions in a shared gene network and/or pathway (Bader et al., 2015; Barreto et al., 2015). This will predict clusters of interacting and functionally related proteins to be misregulated in the hybrids. We detected a protein–protein interaction (PPI) network of 90 genes (34% of the 262 transgressive genes) (Figure 3) with a significant (i.e., more interactions than randomly expected) PPI enrichment (*P* = 4.29 × 10^–2^). We found no evidence of known functional enrichment in the network, but a significant overrepresentation of “Signal” genes based on UniProt keywords (FDR corrected *P* = 1.25 × 10^–7^). More importantly, when we partitioned the network analysis by sterile vs. fertile hybrids, the PPI analysis was significant for sterile hybrids (PPI enrichment *P* = 1.06 × 10^–2^, 79 nodes) but not for fertile hybrids (PPI enrichment *P* = 0.106, four nodes). Twenty-two genes in the network were *cis* or non-compensatory, thus their misregulation could be driven by interactions with other misregulated genes in the network (Figure 3). We found no significant PPI for transgressive genes differentially expressed between subspecies (*P* = 0.597). Finally, we explored whether transgressive expression in hybrids could be a consequence of what we refer to as “transcriptional drive.” That is, the ability of a modified *trans* factors to control and drive the transcription of multiple prior non-target genes with *cis*-sequence similarity (Figure 2A–red arrows). We found 46 genes (18% -46/262) with possible evidence of co-option by newly evolved *trans* mutations. Of these genes, 15 were classified as compensatory, 10 had *cis–trans* divergence, 9 were non-compensatory, and 12 had non-informative SNPs for classification (Supplementary Table 1).

## Discussion

Genome-wide, our results are in agreement with previous reports of correlated evolution between sequence and expression divergence (Castillo-Davis et al., 2004; Nuzhdin et al., 2004; Khaitovich et al., 2005; Lemos et al., 2005; Artieri et al., 2007; Hunt et al., 2013; Whittle et al., 2014; Barreto et al., 2015), but provide no support for positive selection or relaxation of selective constraints as drivers of change causing misregulation and transgressive expression in hybrids. It is possible that the lack of correlation for hybrids results from the relatively low divergence between the subspecies. However, we did find a significant correlation for expression differences between subspecies. Genes with no differential expression between subspecies and transgressive expression in hybrids did not show overall evidence of lower sequence divergence than transgressive genes with differential expression between subspecies. This result is unexpected under a scenario of compensation favoring mutations that restore divergence in gene expression between parental subspecies (i.e., stabilizing selection). We used SNPs to tease apart regulatory divergence among transgressive genes in hybrids. Transgressive expression results from divergence in *cis* and *trans* regulatory elements, leading to differential expression between parental species as well as hybrids. Alternatively, such changes can be buffered by compensatory mutations within lineages to restore levels of expression to similar levels between species but cause misexpression in hybrids (Landry et al., 2005; McManus et al., 2010; Mack and Nachman, 2017). Studies of divergence in gene expression between species provides support for changes in transcript levels being often deleterious, with large mutational effects, and equilibrium levels of genetic variation maintained by stabilizing selection (Rifkin et al., 2003; Lemos et al., 2005; Hodgins-Davis et al., 2015). Our study shows that the majority (79%) of transgressive genes in hybrids between *D. p. pseudoobscura* and *D. p. bogotana* were not differentially expressed between the subspecies, and the SNP analysis supports a good proportion of transgressive expression caused by compensatory changes (49%) during early stages of species divergence, with another (16%) caused by *cis–trans* divergence.

Informative SNPs are limited between closely related subspecies. Thus 25% of transgressive genes could not be analyzed this way. Moreover, for any gene, not all reads have informative SNPs imposing some analytical limitations. While this might lead to an underestimation, our result of 49% compensatory evolution for a pair of very closely related subspecies of *Drosophila* is expected when compared to estimates of 73% compensatory evolution for hybrids between more distantly related species of *D. simulans* and *D. sechellia* (Coolon et al., 2014) and 67% for yeast (Wang et al., 2015). The proportion of compensatory mutations within lineage (49%) is larger than *cis–trans* divergence between lineages (16%) and suggests that hybrids between closely related taxa might be more vulnerable to a breakdown of co-adaptations within species than misregulation caused by divergent evolution. Two important caveats are that we used one line per subspecies, which might overestimate true divergence between these subspecies by overlooking possible shared polymorphisms, and that there is inherent bias toward a possible overestimate of the role of compensatory evolution when using an ASE approach (Fraser, 2019; Zhang and Emerson, 2019). Therefore, it is important to explore possible alternative explanations for a large proportion of transgressive genes, which could not be explained by *cis–trans* compensation or divergent *cis–trans* evolution.

We found that genes with transgressive expression in hybrids that experienced divergence in regulation between subspecies produced proteins that did not show enrichment for interactions. On the other hand, transgressive genes with no evidence of divergence between subspecies were enriched for protein interactions, particularly for the sterile hybrids. The overrepresentation of protein interactions among transgressive genes may be due to possible tissue or cell type atrophy in the hybrids. However, these two subspecies are very closely related, and previous work have shown no evidence of tissue atrophy in the hybrids (Gomes and Civetta, 2014). Furthermore, we did not find an overrepresentation of underexpressed genes as expected under tissue/cell atrophy. This result suggests that in some cases, misregulation and transgressive expression could be a cascade effect driven by networks of interacting proteins and that such domino effect could work to exacerbate initial incompatibilities in hybrids between early stage diverging lineages. The role of gene-network effects is expected under the Bateson–Dobzhansky–Muller model of speciation (Turner et al., 2014), and while there has been some support for gene-networks buffering allelic variation among yeast strains (Bader et al., 2015), its importance in speciation is largely unexplored. Finally, we entertained the idea that newly arising *trans* mutations in either divergent or compensatory cases could possibly generate a cascade effect of misregulation of targets that might have not experienced *cis*-regulatory mutations between divergent taxa (Figure 2A–red arrows). We explored the idea of “transcriptional drive by sequence similarity among targets” by seeking sequence similarity within proximal (2,000 bp) putative *cis*-regulatory elements between transgressive genes showing evidence of *cis–trans* divergence or compensation and those showing no evidence of such sequence divergence. Our analysis showed some support for this idea with 18% of genes being possibly co-opted. However, only nine genes classified as non-compensatory appear as possible targets. One important limitation is that we only addressed sequence similarities between nearby upstream sequence regions of compensatory or *cis–trans* transgressive genes and upstream sequence regions of other transgressive genes, leaving unexplored the possibility that misregulation could be exerted by more distant *cis*-regulatory elements.

## Data Availability Statement

Publicly available datasets were analyzed in this study. This data can be found here: *Drosophila bogotana* SRA at GenBank: SRX091468. *D. pseudoobscura* extracted from FlyBase (http://flybase.org/). Expression data and other data can be accessed on Dryad (10.5061/dryad.2z34tmpjv).

## Author Contributions

AC designed the study. AG conducted the data analysis. Both authors contributed to writing and editing the manuscript.

## Conflict of Interest

The authors declare that the research was conducted in the absence of any commercial or financial relationships that could be construed as a potential conflict of interest.

## Footnotes

1 http://flybase.org/

2 http://usegalaxy.org
