# Supplementary material for: Hybrid Incompatibilities and Transgressive Gene Expression Between Two Closely Related Subspecies of Drosophila
Source: Front Genet. 2020 Dec 10;11:599292. doi: 10.3389/fgene.2020.599292 (PMC7758320; doi:10.3389/fgene.2020.599292)
Supplement: Supplementary file 1 [file Data_Sheet_1.docx]

**Supplementary Results**

*Limited differential expression between subspecies and few genes with transgressive expression in hybrids*

Approximately 290 million reads were mapped to the *D. p. pseudoobscura* reference genome. We checked for potential mapping bias against *D. p. bogotana* but found that *D. p. bogotana* and *D. p. pseudoobscura* had very similar percentages of total RNA sequence reads mapped (83.54% and 82.11% respectively). Sterile and fertile hybrids also had similar proportions of mapped reads to the reference genome (85.09% and 82.95% respectively). Moreover, out of a total of 16,726 genes annotated, we did not find an overrepresentation of genes with higher average expression in *D. p. pseudoobscura* than *D. p. bogotana* (8,944 and 8,621 respectively), which would have been expected if there was biased mapping.

Under the more stringent lfc threshold of 1, only 819 genes were differentially expressed between the parental subspecies (4.9% of annotated genes), with equal proportions of genes with higher expression in one species or the other (398 in *D. p. bogotana vs.* 421 in *D. p. pseudoobscura*). A limited number of genes showed transgressive expression in hybrids (44) with a significantly higher proportion in the sterile F_1_ hybrid males (39) than fertile F_1_ hybrid males (4) (Z = 7.5; *P*<0.00001). One gene showed transgressive expression in both sterile and fertile hybrids. Using the less stringent threshold of lfc 0.5, the number of differentially expressed genes between the parental subspecies increases to 2,179 (13.03% of total annotated genes; Figure S1). The proportion of genes with higher expression in one species than the other remains similar with 1,103 genes with higher expression in *D. p. bogotana* *vs.* 1,076 in the *D. p. pseudoobscura*. The trend of a few genes showing transgressive expression in the hybrids and a higher proportion of transgressive genes in the sterile hybrids relative to the fertile hybrids remains the same with a lfc threshold of 0.5. Of the 262 transgressive genes between the hybrids, a significant proportion belonged to the sterile F_1_ hybrids (240) (Z = 18.4, *P*<0.00001), while only 18 genes showed transgressive expression in the fertile hybrids. Four genes had transgressive expression in both hybrids.

**Figure S1**. Differential gene expression between the two parental subspecies. DESeq2 normalised counts were used as a measure of gene expression. The expression of 16,726 genes were measured. Genes that are differentially expressed between subspecies are shaded black and red while those that do not show differential expression are grey. Circles denote protein coding genes and non-protein coding genes are represented by triangles. Under the less stringent log_2_-fold change threshold of 0.5, 2,179 (303 non-coding) genes were differentially expressed between subspecies.


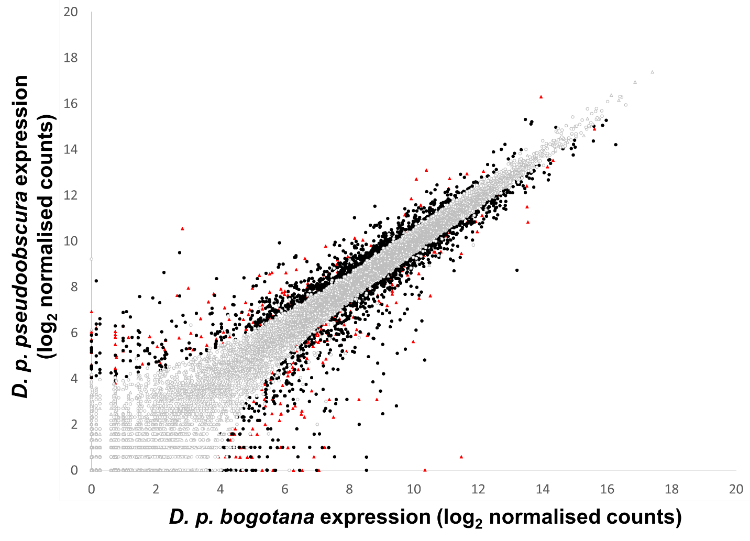


**Table S1**: BLASTn results using compensatory and *cis-trans* transgressive genes as queries. For Matches, black FBgn= compensatory genes, black underlined= non-compensatory; blue= *cis-trans*; blue underlined= *cis*; and grey= NA. % Id= Percentage identity. GWH= Genome-wide hits; “Unique” if no hits other than the ones shown in matches, otherwise the best hits (lowest e-value) is shown.

| Query | Matches | E-values | % Id | Other GWH |
| --- | --- | --- | --- | --- |
| FBgn0070459 | FBgn0271996 | 0 | 100 | Unique |
| FBgn0072286 | FBgn0077417; FBgn0079358 FBgn0248786; FBgn0271117 FBgn0271521; FBgn0272358  FBgn0273410 | 9e-20; 6e-29  7e-28; 3e-20  2e-34; 7e-28  1e-23 | 98; 88  86; 94  91; 86  88 | Unique |
| FBgn0078355 | FBgn0071944; FBgn0078943  FBgn0079731; FBgn0080577  FBgn0245144 | 1e-23; 4e-49  5e-17; 2e-41  2e-41 | 73; 77  72; 80  78 | Unique |
| FBgn0078546 | FBgn0074132; FBgn0247626 | 2e-30; 4e-58 | 75; 85 | FBgn0077122 (2e-07)  FBgn0078535 (2e-07) |
| FBgn0078680 | FBgn0071944; FBgn0075542  FBgn0078943; FBgn0080577  FBgn0081015; FBgn0244760  FBgn0245144 | 5e-33; 2e-51  6e-64; 3e-43  3e-30; 5e-33  4e-47 | 77; 79  85; 84  73; 74  82 | Unique |
| FBgn0079637 | FBgn0071944  FBgn0075000; FBgn0077499  FBgn0078943; FBgn0080577  FBgn0244760; FBgn0245851 | 6e-17  9e-27; 2e-15  8e-15; 8e-15  4e-25; 1e-18 | 72; 74  64; 71  70; 71  75; 70 | FBgn0080782 (6e-13) |
| FBgn0079731 | FBgn0071944 | 1e-18 | 75 | FBgn0272290 (4e-08) |
| FBgn0245605 | FBgn0071718; FBgn0077417  FBgn0247977 | 4e-75; 3e-26  3e-96 | 78; 97  74 | Unique |
| FBgn0248096 | FBgn0071718 | 9e-20 | 81 | Unique |
| FBgn0250421 | FBgn0079358; FBgn0248786  FBgn0271521; FBgn0273410 | 2e-26; 6e-33  1e-35; 2e-21 | 87; 76  92; 87 | FBgn0271418 (6e-04) |
| FBgn0262055 | FBgn0271812 | 0 | 100 | Unique |
| FBgn0271245 | FBgn0246515; FBgn0250150  FBgn0250421 | 2e-34; 6e-34  2e-21 | 82; 80  81 | FBgn0081107 (2e-05) |
| FBgn0271910 | FBgn0272900 | 2e-160 | 100 | Unique |
| FBgn0272358 | FBgn0079358; FBgn0248786  FBgn0271521 | 4e-23; 0  4e-24 | 90; 100  85 | Unique |
